# Editorial: Nanotechnology-based delivery systems for cancer treatment

**DOI:** 10.3389/fbioe.2026.1785087

**Published:** 2026-01-23

**Authors:** Alicia Fernandez-Fernandez

**Affiliations:** Dr. Kiran Patel College of Osteopathic Medicine, School of Rehabilitative Sciences, Physical Therapy Department, Nova Southeastern University, Fort Lauderdale, FL, United States

**Keywords:** cancer, immune response, nanocarrier (nanoparticle), nano-immunotherapy, nanotechnology, nanotherapeutic formulation

Nanotechnology-based systems have become an important pillar of contemporary cancer therapeutics, offering ways to improve drug exposure in tumors while reducing systemic toxicity and allowing for theranostic applications in cancer management (Giri et al., 2023; Pallares et al., 2025). Nano-formulations, including liposomes, polymeric micelles, inorganic nanoparticles, hydrogels, cell-based carriers, and others, are increasingly being designed in a more tailored manner that considers tumor biology and immune context (Abdullah et al., 2025). Although challenges to clinical translation remain, these innovations beyond simple drug encapsulation represent a crucial opportunity for nanotechnology approaches (Bhatia et al., 2022; Wang et al., 2025). Advances in stimuli-responsive platforms, which can be triggered by pH, redox state, enzymes, or light, have enabled researchers to more finely control spatial and temporal release for both small molecules and gene therapies (Zhao et al., 2021). Nanomaterials can also leverage immune responses to reshape the tumor microenvironment, enhance antigen presentation, and improve performance of checkpoint inhibitors and cancer vaccines (Fallatah et al., 2025; Wang et al., 2025), making immunotherapy-related formulations a promising tool in the field of cancer nano-therapy. Against this background, the current Frontiers Research Topic “Nanotechnology-Based Delivery Systems for Cancer Treatment” offers a focused snapshot of new advances in immune-active, precision-oriented nano-platforms.

Several contributions in this collection specifically explore how nanotechnology can deepen and broaden immune responses. The mini-review “Research advances in immune agonists and their nanoparticles for enhancing the immunotherapeutic efficacy of PD-1 inhibitors in malignancies” discusses how immune agonist nanoparticles are capable of enhancing tumor-specific accumulation, prolonging agonist half-life, and synergizing with PD-1 inhibitors to remodel immunosuppressive microenvironments. By tuning size, charge, and stimuli-responsiveness, these nanocarriers concentrate immune agonists in tumors, enhance T-cell infiltration, and help convert immunologically “cold” lesions into “hot” ones that respond better to PD-1 blockade. Complementing this overview, the review “Opportunities, obstacles and challenges of nano-immunotherapy in melanoma” maps how nanoparticles can impact melanoma by reshaping the tumor immune microenvironment, improving checkpoint inhibitor responses, enhancing immune activation, and enabling rational combinations of photothermal or photodynamic therapy with immunotherapy in a way that is increasingly compatible with clinical practice. This review also provides an assessment of current application challenges, and a clear overview of advantages and disadvantages of different nanoparticle types based on their *in vivo* properties such as biocompatibility, biodegradability, stability, and immunomodulation. The mini-review “Reprogramming the tumor-immune landscape via nanomaterial-induced immunogenic cell death” further extends the theme of interlinked nanomaterial and immune actions with a mechanistic focus, discussing why nanomaterials are ideal for inducing immunogenic cell death (ICD), and recent notable examples of this approach. For instance, they highlight nanoplatforms that actively trigger immunogenic cell death through reactive oxygen species generation, hypoxia modulation, and acidity correction; boosting dendritic-cell activation and cytotoxic T-cell priming when combined with established immunotherapies such as checkpoint inhibitors and CAR-T cells. Together, these three articles present an optimistic and cohesive vision of nano-immunotherapy as a practical strategy for enhancing therapeutic response in resistant tumors.

The research topic also showcases original research that demonstrates how engineered nanocarriers can facilitate potent interactions between therapy and systemic immunologic responses. The osteosarcoma study “Manganese-pyrochloric acid photosensitizer nanocomplexes against osteosarcoma: achieving both high activatability and high effectiveness” introduces Mn^2+^-pyrochloric acid nanocomplexes that co-deliver photosensitizer and metal ion to bone tumors, achieve fluorescence recovery and on-demand photodynamic activation, and simultaneously engage the cGAS-STING pathway via released Mn^2+^ to elicit robust dendritic-cell maturation and CD8^+^ T-cell responses. This approach essentially couples local photodynamic therapy with systemic effects. A related immuno-photothermal strategy is demonstrated in “Mesoporous silica nanoparticles loaded Au nanodots: a self-amplifying immunotherapeutic depot for photothermal immunotherapy,” where mesoporous silica provides high loading capacity and controlled release, while Au nanodots enable mild photothermal heating that both kills tumor cells and enhances immunogenic signaling, amplifying local and systemic antitumor immunity. This approach shows that mild photothermal therapy can be combined with strong induction of immunogenic cell death and T-cell activation, effectively expanding the impact of local heat treatment into potential systemic effects. On the delivery-systems side, the systematic review “Hydrogels in cancer treatment: mapping the future of precision drug delivery” uses bibliometric and content analysis to chart how hydrogel-based formulations, often hybridized with nanoparticles or immunomodulators, are emerging as adaptable, minimally invasive platforms for localized and sustained drug release across different cancer types. It also identifies nano-composite hydrogels and immunotherapy-loaded hydrogels as particularly promising future directions.

Finally, two more articles in the topic explore how nanotechnology can safely interface with complex cellular systems, emphasizing the importance of understanding and leveraging the actions and effects of nanoformulations in conjunction with existing biological pathways and processes. In “DNA-loaded targeted nanoparticles as a safe platform to produce exogenous proteins in tumor B cells,” the authors demonstrate that antibody-targeted nanoparticles can deliver DNA to malignant B cells and drive controlled expression of exogenous proteins, while maintaining a favorable safety profile both *in vitro* and *in vivo*. This suggests an attractive, non-viral approach for *in situ* protein or gene delivery that could synergize with emerging B-cell–directed immunotherapies. The manuscript “Loading monocytes with magnetic nanoparticles enables their magnetic control without toxicity” shows that human monocytic cells can safely internalize citrate-coated or gold-coated superparamagnetic iron oxide nanoparticles and remain viable with minimal induction of reactive oxygen species. They can then be magnetically steered and enriched *in vitro*, highlighting the feasibility of exploring magnetically guided cell-based drug or gene delivery in future works.

Collectively, these studies bring together complementary views of biological modes of action: immune agonism, immunogenic cell death, local therapies with systemic effects, cell-assisted targeting, and novel formulation approaches that emphasize interactions with complex cells and systems. While the overall outlook is optimistic, the topic articles also touch upon existing challenges such as immunotoxicity and scale-up. To achieve successful clinical translation, nano-delivery systems must emphasize consistent characterization, scalability, and customization to specific biomarkers (Bhatia et al., 2022; Tong et al., 2024); and these are questions that researchers should be asking in the years to come to facilitate the incorporation of nano-approaches into routine cancer care. We hope that this collection of articles provides the reader with an overview of recent advances in the field, and ideas for further development.
